# Reply to: Does the KDEL receptor cycle between the Golgi and the ER?

**DOI:** 10.1038/s41467-024-45850-7

**Published:** 2024-03-20

**Authors:** Jurgen Denecke

**Affiliations:** Centre for Plant Sciences, School of Biology, Faculty of Biological Sciences, University of Leeds, Leeds, LS2 9JT UK

**Keywords:** Golgi, Protein transport

**replying to** F. Aniento & D. G. Robinson *Nature Communications* 10.1038/s41467-024-45849-0 (2024)

We have previously shown that the ability of the K/HDEL receptor (ERD2) to mediate the accumulation of K/HDEL proteins in the endoplasmic reticulum (ER) depends on two conserved Leucine residues near the C-terminus, which also mediate ERD2 Golgi localisation^1^. We have now shown that the di-leucine signal does not promote ER export, but instead prevents Golgi to ER recycling of ERD2, contributing to its Golgi-retention^2^. The signal is conserved between plants and humans, and when obstructed by a C-terminally fused fluorescent protein, the fusion protein can be re-activated by an alternative Golgi-retention motif. These and many other new results^2^ forced us to abandon the popular recycling model. In their Matters Arising, Aniento and Robinson take issue with our approach of visualising ERD2^1^, but do not acknowledge the most important recent findings^2^.

## The origins of the recycling model

Over 3 decades ago, the cell biology community learned that protein secretion occurs by default, but it can be prevented by signals for transport to lysosomes/vacuoles^3,4^ or accumulation in the ER^5^. The search for the underlying sorting receptors first led to the discovery of Mannose 6 phosphate (M6P) receptors. Abundant enough to be affinity-purified, they also turned out to recycle between the Golgi apparatus and post-Golgi organelles^6^. The discovery of the K/HDEL receptor was much harder and required a brilliant genetic screen to select for mutant yeast cells unable to retain an HDEL-tagged protein in the ER^7^. We, therefore, use the genetic annotation ER retention defective 2 (ERD2) to describe the K/HDEL receptor.

Visualising ERD2 by C-terminal fusion to the c-myc epitope (and later fluorescent proteins) revealed a dual ER-Golgi distribution that could be shifted further to the ER when ligands are overexpressed^8^. This was widely seen as confirmation that ERD2 recycles like other receptors. We and others based our work on this model, until unexpected results led us to think again^1,2^. A model is never more than an attempt to explain a process that escapes direct observation. It cannot be proven unless a technical advance allows said observation. However, a single inconsistency should suffice to question, re-consider, modify, or even reject a model, regardless of its popularity.

## ERD2 function depends on a native C-terminus

Our rationale for this topic has been both pragmatic and self-critical. Measuring an ERD2-dependent increase in the retention of HDEL cargo is the most direct way to monitor ERD2 activity, as it follows the cause (ERD2) and consequence (better ER retention) principle. Using our sensitive and reproducible ERD2 dose-response assay^1^ we were disappointed to find that ERD2-YFP failed to validate when directly compared with untagged ERD2^1^. A systematic set of experiments revealed that C-terminally fused YFP masks a conserved di-leucine signal that is responsible for both the activity and the Golgi localisation of ERD2^1^.

In a recent follow-up paper^2^, we ruled out that the di-leucine signal mediates rapid ER export, but instead prevents Golgi to ER recycling of ERD2. Our biologically active fusion YFP-TM-ERD2^1^ also passed two independent genetic validation tests^2^, whilst ERD2-YFP failed. Aniento and Robinson have not engaged with these findings, reiterated their earlier concerns^9^ about our R/YFP-TM-ERD2 fusions^1^ and continue to defend the use of C-terminal ERD2 fusions, claiming that our findings contradict the author’s earlier data with ERD2-YFP^10^. In fact, there is no contradiction, because the authors did not directly compare native ERD2 with ERD2-YFP within the same protoplast transfection batches, as we did^1^. It is possible that in their expression systems, ERD2-YFP was expressed at higher levels and then displayed a very weak activity. Indeed, high expression favours Golgi localisation of ERD2-YFP whilst low expression favours ER localisation, even without co-expressed ligands^2^. This alone should raise alarm bells for any ERD2-YFP redistribution assays when expression levels are not normalised^11,12^.

Our dose-response assays illustrate the true difference in performance between native and tagged ERD2. For instance, ERD2-myc or ERD2-FLAG do have a weak residual ligand-sorting activity^2^, but they require 10-fold higher synthesis levels to match the activity of untagged native ERD2. This means the C-terminal epitope fusions have lost 90% of their activity. In fact, our results^2^ are perfectly comparable to results in mammalian cells showing equally weak ERD2-myc activities^13,14^, and since these studies did not directly compare ERD2-myc with native ERD2 either, there is no contradiction at all. We maintain that our combined results^1,2^ strongly imply staying away from the C-terminus and encourage the field to come to terms with that.

## The problem of circular evidence

ERD2 redistribution to the ER has been used as a replacement for a direct K/HDEL cargo sorting assay since 1992. We can also readily reproduce ligand-induced ERD2-YFP redistribution to the ER^2^. The active YFP-TM-ERD2 fusion persists at the Golgi but behaves like ERD2-YFP when the last 5 amino acids (LQLPA) harbouring the Golgi-retention signal are deleted^2^. This suggests that both ERD2-YFP as well as YFP-TM-ERD2 can specifically interact with ligands, change conformation and may be dragged to the ER. But to see this with YFP-TM-ERD2, we have to delete the Golgi-retention signal first. This is why we do not consider the redistribution assay biologically relevant. We have the same concerns when C-terminal ERD2 fusions have been used to document ERD2 oligomerisation, interactions with COPI components, ARF1 or P24 proteins^10,15,16^ as these observations may have materialised due to an exacerbated ERD2 recycling back to the ER.

In short, the redistribution assay is an example of circular evidence, the assumption that all sorting receptors must recycle and a second assumption stating that ERD2-YFP (or ERD2-myc) has normal biological activity. Our direct comparison of such fusions with native ERD2 shows that the second assumption is wrong. We also argue that the unique position of ERD2 in the pathway, mediating the return of proteins to where they originally came from (the ER), renders eternal recycling a futile strategy, referred to as the Sisyphus paradox^2^.

It must be noted that the ligand-induced redistribution assay should not be confused with the Brefeldin-A-like effect caused by ERD2 overexpression (rather than ligand overexpression)^17^. We have observed this too, illustrated by the formation of the ER-Golgi supercompartment and a reduction in constitutive secretion^2^. Deletion of the last 5 amino acids harbouring the di-leucine Golgi-retention signal abolishes the BFA effect^2^. ERD2-YFP partitioning to the ER at low expression levels^2^ has therefore nothing to do with the BFA effect, also because the di-leucine signal is masked in this construct^1^.

## Golgi retention is essential for ERD2 function

Experimental results impossible to reconcile with the recycling model are the demonstration that the di-leucine motif is (1) essential for ERD2 activity defined by its ability to sort ligands^1^, that it specifies (2) Golgi-retention rather than rapid ER export^2^, and that (3) the introduction of an alternative Golgi-retention signal^18^ can reactivate ERD2-YFP. These results fully supersede any issues raised in a previous review^9^ and have not been acknowledged by the Matters Arising.

In addition, if COPI-mediated ERD2 recycling based on lysine residues were important for ERD2 function, then mutating these residues in native, untagged ERD2 should have been fatal for its ability to retain HDEL proteins. These mutations had no effect on plant- or human ERD2^1,2^. Even the evidence for the role of lysines in the redistribution of C-terminal ERD2 fusions is divided. One study attributed a role of these lysines in the redistribution of C-terminally tagged fluorescent ERD2^11^, but an earlier study with ERD2-myc did not^19^. Perhaps these differences were entirely due to variances in expression levels, which we have demonstrated to have a significant influence alone^2^. Finally, di-lysine motifs were shown to be extremely sensitive to C-terminal extensions with just one or two serines^20^. The c-myc tag, and certainly an entire fluorescent protein (with or without linker), would pose an even larger hurdle for those lysines to interact with COPI machinery.

## Final considerations

It should not be categorically ruled out that a small amount of ERD2 does indeed recycle and may get dragged to the ER during ligand overload, but when the LQLPA (LSLPA in human ERD2) sequence is unobstructed, we cannot detect it (yet). Two separate studies reported an apparent increase in diffuse staining of endogenous untagged ERD2 when overloaded with ligands^12,21^. The earlier study carefully stated that the increased diffuse staining pattern could not categorically be interpreted as ER^21^. It is also likely that ligand overload by multicopy expression was much higher in these mammalian cell models compared to plant cells. No retention signal is perfect, and detection of untagged ERD2 in transit through the ER may occur upon severe ligand overload. But this does not take away that ERD2 contains a Golgi-retention signal to prevent this^2^, and a direct comparison of ERD2 with and without this signal demonstrates that ERD2 retention in the Golgi is essential for its function. The field should define ERD2 function by its ability to increase the HDEL-protein retention capacity^1,2^, rather than how ligands influence ERD2.

The problem with C-terminal fusions will certainly not go away, and if other groups directly compare native ERD2 with ERD2-YFP (or ERD2-myc) they will doubtlessly see the same results, as long as they have an internal reference gene to achieve comparable transfection rates to allow a fair comparison. It is also necessary to carry out dose-responses because at high expression the difference becomes less obvious. The limitation of our work is that whilst our ligand-sorting assays are carried out with untagged ERD2 and mutants thereof, our subcellular localisations still require fluorescent tagging. Due to the very low abundance of endogenous ERD2 in plant cells, this challenge may be difficult to overcome, but we are working on it.
